# Metagenomic analysis reveals potential interactions in an artificial coculture

**DOI:** 10.1186/s13568-017-0490-2

**Published:** 2017-11-02

**Authors:** Minglei Ren, Guiying Zhang, Zi Ye, Zhixian Qiao, Meili Xie, Yan Lin, Tao Li, Jindong Zhao

**Affiliations:** 1 State Key Laboratory of Freshwater Ecology and Biotechnology, Institute of Hydrobiology, Chinese Academy of Sciences, Wuhan, 430072 China; 2 College of Life Science, Peking University, Beijing, 100871 China; 3 Present Address: Simon F. S. Li Marine Science Laboratory, School of Life Sciences, The Chinese University of Hong Kong, Shatin, Hong Kong SAR China; 4 Present Address: Southern University of Science and Technology, Shenzhen, 518055 China

**Keywords:** Biosynthesis of vitamin B_12_, Compatible solute, Fatty acid, Metagenome, *Synechococcus*, *Mesorhizobium*, *Microcystis*

## Abstract

Disentangling the interactions between cyanobacteria and associated bacterial community is important for understanding the mechanisms that mediate the formation of cyanobacterial blooms in freshwater ecosystems. Despite the fact that a metagenomic approach enables researchers to profile the structure of microbial communities associated with cyanobacteria, reconstructing genome sequences for all members remains inefficient, due to the inherent enormous microbial diversity. Here, we have established a stable coculture system under high salinity, originally from a mixture of an axenic cyanobacterium *Synechococcus* sp. PCC 7002 and a non-axenic bloom-forming cyanobacterium *Microcystis* colony. Metagenomic analysis showed that the coculture consists of *S.* sp. PCC 7002 and two heterotrophic bacteria, designated as *Pseudomonas stutzeri* TAIHU and *Mesorhizobium* sp. TAIHU, respectively. And near-complete genome sequences of both bacteria were reconstructed from the metagenomic dataset with an average completeness of 99.8%. Genome-wide pathway analysis revealed that *M.* sp. TAIHU carried all the genes involved in the de novo biosynthesis of cobalamin, which is required by *S.* sp. PCC 7002 for growth. To cope with the high salinity in the coculture, experimental evidence demonstrated that *S.* sp. PCC 7002 would synthesize the compatible solutes including sucrose and glucosylglycerol, which are supposed to be exploited by both heterotrophic bacteria as potential carbon and/or nitrogen sources. Furthermore, the genes encoding for the biosynthesis of the ectoine, another common osmolyte are found exclusively in *P. stutzeri* TAIHU, while the genes responsible for the catabolism of ectoine and its derives are present only in *M.* sp. TAIHU. These genomic evidence indicates beneficial interaction between three members in the coculture. Establishment of the coculture system with relative simplicity provides a useful model system for investigating the interspecies interactions, and genome sequences of both bacteria associated with *Microcystis* bloom described here will facilitate the researcher to elucidate the role of these heterotrophic bacteria in the formation and maintenance of cyanobacterial bloom in freshwater ecosystem.

**Electronic supplementary material:**

The online version of this article (doi:10.1186/s13568-017-0490-2) contains supplementary material, which is available to authorized users.

## Introduction

Among the blooms produced by genera of the cyanobacteria, *Microcystis* blooms might be the most widely spread (Harke et al. 2016). Most *Microcystis* synthesize and exude toxic secondary metabolites including microcystins, into water environments, causing intoxication of humans and animals (Sivonen and Jones 1999; Bláha et al. 2009). To understand the formation mechanism of *Microcystis* blooms, various associated biotic and abiotic factors have been studied extensively (Shapiro 1973; Jacoby et al. 2000; Paerl et al. 2001; Oliver and Ganf 2002), especially the roles and diversity of the associated heterotrophic bacteria in the mucilaginous colonies formed by *Microcystis*. The interactions between *Microcystis* and bacteria, are also areas of research interest, including the degradation of secondary metabolites, the inhibitory or enhancing effects on the growth of the cyanobacteria, resource competition, and nutrient exchange (Cole 1982; Fuks et al. 2005; Berg et al. 2008; Lemes et al. 2008; Dziallas and Grossart 2011; Shen et al. 2011; Dziallas and Grossart 2012; Briand et al. 2016; Zhu et al. 2016).

Before the advent of the second generation sequencing technology, traditional molecular techniques, including denaturing gradient gel electrophoresis (Muyzer et al. 1993), terminal restriction fragment length polymorphism analysis (Tiedje et al. 1999), and the analysis of conserved marker genes, e.g. 16S rRNA genes (Sogin et al. 2006; McHardy and Rigoutsos 2007), have been applied to profile the diversity of heterotrophic bacteria associated with cyanobacteria (Eiler and Bertilsson 2004; Kolmonen et al. 2004). As a cultivation-independent method, metagenomics provides a way of characterizing the uncultured microbes from their natural habitats in a higher resolution (Mick and Sorek 2014), and also enables the researcher to understand the potential role of these microbes in the community. Consequently, the microbial consortia from various environments, such as ocean water, natural acidophilic biofilms, permafrost, acetate-amended aquifers, and activated sludge bioreactors, have been studied using the metagenomic approach, leading to a better understanding of microbial and functional diversity, the discovery of novel genes, and the reconstruction of genomes from the microbial community (Tyson et al. 2004; Venter et al. 2004; Mackelprang et al. 2011; Wrighton et al. 2012; Albertsen et al. 2013). Metagenomics has also been applied to the study of cyanobacterial blooms (Pope and Patel 2008; Li et al. 2011; Steffen et al. 2012; Mou et al. 2013), showing that *Microcystis* bloom samples contained an enormous range of heterotrophic bacteria (Li et al. 2011; Steffen et al. 2012). Evidence from the study of long-term laboratory *Microcystis* colonies revealed that the associated bacteria played an important role in the degradation of benzoate and biosynthesis of vitamin B_12_ (Xie et al. 2016). These works have provided valuable insight into the molecular mechanisms that underpin the interactions between *Microcystis* and heterotrophic bacteria in the colony.

Despite its great potential in the analysis of microbial communities, metagenomic approach still has limitations in disentangling the relationships between each organism. The great diversity of bacteria in the sample makes it difficult to obtain all bacterial genomes with high completeness, causing the deficiency of genome information to investigate the interaction between *Microcystis* and associated bacteria (Li et al. 2011; Steffen et al. 2012). Although the typical approach of isolation followed by cocultivation was often adopted to study the interaction between community members (Shen et al. 2011), isolating associated bacteria and selecting the proper growth media seems time-consuming and tedious (Berg et al. 2008). To study the nature of interactions between bacteria, it is an alternative approach to establish an artificial coculture system with limited number of bacteria species in the laboratory (Amin et al. 2009; Kazamia et al. 2012). Sequencing of a simpler coculture system would lead to a higher completeness of genome sequence from species. It’s also easier to study the potential relationships between community members (Cole et al. 2014; Xie et al. 2016). Compared with samples collected directly from *Microcystis* bloom in the natural environment, *Microcystis* colony which maintains in the laboratory for a long term, encompass a limited number of heterotrophic bacteria (Xie et al. 2016; Zhu et al. 2016). Although a coculture system cannot represent the entire microbial diversity in a natural community, the insight gained from the analysis of the coculture could help to develop a fundamental understanding of the interaction between community members in nature.

*Synechococcus* sp. PCC 7002 is a unicellular cyanobacterium, which was originally isolated from an inshore marine mud sample in Puerto Rico (Baalen 1962). And it can grow over a wide range of NaCl concentration in the laboratory (Batterton and Baalen 1971). In addition, the complete genome sequence of *S.* sp. PCC 7002 has been determined (see http://www.ncbi.nlm.nih.gov/) and it can be easily genetically transformed (Stevens and Porter 1980; Frigaard et al. 2004), making it one of the most well-studied model cyanobacteria. Here we have established a coculture system consisting of *S.* sp. PCC 7002 and heterotrophic bacteria associated with *Microcystis*. The stable coculture system is characterized by high salinity and potential symbiotic interactions between *S.* sp. PCC 7002 and cobalamin-producing bacteria. The composition of the coculture and the producer of cobalamin, which is required by *S.* sp. PCC 7002 for growth, were determined through metagenomic analysis. Our results uncovered a potential symbiotic interaction between *S.* sp. PCC 7002 and *Mesorhizobium* sp. TAIHU in the coculture. Genes responsible for transporting cobalamin and encoding cobalamin-dependent enzymes were also investigated and compared between individual members in coculture. The approach of establishing a coculture system with symbiosis in our study will facilitate further studies of the relationship between cyanobacteria and its associated bacteria.

## Materials and methods

### Strain sources and coculture establishment

*Microcystis aeruginosa* TAIHU98 strain (FACHB-1752) (Yang et al. 2013, 2015) was obtained from the Freshwater Algae Collection of the Institution of Hydrobiology, Chinese Academy of Sciences (FACHB collection), Wuhan, China. The strain, initially collected from Lake Taihu in 2006, was part of a colony comprising other bacteria. Initially, we cocultured the *Microcystis* colony with an axenic *Synechococcus* sp. PCC 7002. At the beginning of the experiment, 500 µl liquid culture of axenic *S.* sp. PCC 7002 and 500 µl liquid culture of *Microcystis* colony were transferred together into 100 mL autoclaved BG11 medium (Stanier et al. 1971). The coculture was maintained at 25 ± 1 °C, and illuminated in a 12:12 h light:dark cycle with irradiance of 50 µEm^−2^ s^−1^. During subculture, different concentrations of NaCl solution were applied to the BG11 medium in a step gradient (0, 18, 24, 48, 60, 90, and 96 g l^−1^ NaCl). Previous salt-shock experiments revealed that *M. aeruginosa* PCC 7806 endured temporary salinity as high as 17.5 g l^−1^ (Tonk et al. 2007), while the euryhaline cyanobacterium *S.* sp. PCC 7002 grow over a wide range of NaCl concentration (Batterton and Baalen 1971; Ludwig and Bryant 2012). The final concentration of NaCl in the medium was then adjusted to 60 g l^−1^, at which the coculture was stable. The mixture of cells was harvested at the exponential phase of algae cell (OD_730nm_ = 1.5) for sequencing (Ludwig and Bryant 2012).

### Growth curve and SEM image of the coculture

As a comparison, axenic *Synechococcus* sp. PCC 7002 was also maintained in BG11 medium with exogenously added vitamin B_12_ and 60 g l^−1^ NaCl. The final concentration of vitamin B_12_ was the same as the concentration in A plus medium (Ludwig and Bryant 2012), which is used to cultivate *S.* sp. PCC 7002 in the laboratory. The optical density at 730 nm was measured to monitor the cell growth in axenic and coculture conditions. Scanning electron microscopy (SEM) for the two conditions was performed at the Wuhan Institute of Virology, Chinese Academy of Sciences, Wuhan, China. Samples for the SEM operation were prepared using a published method (Gong et al. 2005).

### Sequencing and data cleaning

The algae cells from coculture in the exponential stage were collected for sequencing. Total DNA of the coculture sample was extracted using a previous method (Yang et al. 2013). The sample was sequenced on an Illumina MiSeq sequencer using paired-end technology (301 bp) with the library insert size being 370 bp. To reconstruct the genome sequences of heterotrophic bacteria, the coculture sample was sequenced in another run using MiSeq with library size 420 bp. The library construction and sequencing were conducted in the Institution of Hydrobiology, Chinese Academy of Sciences, according to the manufacturer’s protocol (http://www.illumina.com). Quality control was performed using cutadapt v.1.8.0 (Martin 2011). All the paired reads were merged by FLASH (Magoc and Salzberg 2011) to obtain longer reads. Reads from the coculture sample were pooled together to perform de novo assembly of the coculture sequences.

### De novo assembly and contigs binning

De novo assembly was conducted using SPAdes (Bankevich et al. 2012), Velvet (Zerbino and Birney 2008), and IDBA-UD (Peng et al. 2012), respectively. The optimal assembly was selected based on the typical assembly assessment metrics, including N50 [the N50 is defined as the shortest sequence length at 50% of the contigs or genome (Earl et al. 2011)], total size, the largest contigs length and the number of contigs. Different binning strategies were utilized to reconstruct the genome sequences of heterotrophic bacteria from the mini-metagenomic dataset. The binning process was performed using our previous binning method (Xie et al. 2016) combined with the Blobology (Kumar et al. 2013) and MaxBin methods (Wu et al. 2014). The binning result was evaluated further through alignment similarity using BLASTN (Altschul et al. 1997), and some unassigned scaffolds were recruited into individual genome bins according to taxonomy at the family level.

### Phylogenetic analysis

Before reconstructing genome sequence of individual from the coculture, 16S rRNA genes were predicted from both the clean reads and assembly results, using ÉMIGRÉ (Miller et al. 2011) and RNAMmer (Lagesen et al. 2007), respectively. To further determine the taxonomic location of strains from the coculture, a comprehensive phylogenetic tree was built based on the alignment of 16S rRNA sequences of all species in the same genus with available draft or complete genome sequences. The genome sequences of 23 *Pseudomonas stutzeri* strains were downloaded from *Pseudomonas* database (Winsor et al. 2016), and two strains with partial 16S rRNA were discarded. Genome sequences of 81 strains belonging to the genus *Mesorhizobium* were downloaded from NCBI in December 2015. The phylogenetic trees were constructed using MEGA6 (Tamura et al. 2013). The genome-to-genome distances, which were used to mimic DNA–DNA hybridization (DDH) (Meier-Kolthoff et al. 2013), and the average nucleotide identity (ANI) (Richter and Rossello-Mora 2009; Chan et al. 2012), were calculated to delineate the bacteria in coculture.

### Genome annotation and metabolic pathway analysis

Genome annotation of *Pseudomonas stutzeri* TAIHU and *Mesorhizobium* sp. TAIHU in the coculture was performed using the Prokka program (Seemann 2014) and RAST (Aziz et al. 2008), respectively. Open reading frame sequences predicted by Prokka were annotated by BlastKOALA, an online genome annotation service provided by the Kyoto Encyclopedia of Genes and Genomes (KEGG) (Kanehisa et al. 2015). Pathway analysis was performed mainly by the “Reconstruct pathway” function of the KEGG mapper, and simultaneously combined with the subsystem function in the RAST server.

## Results

### Characterization of the coculture system

**Fig. 1:**
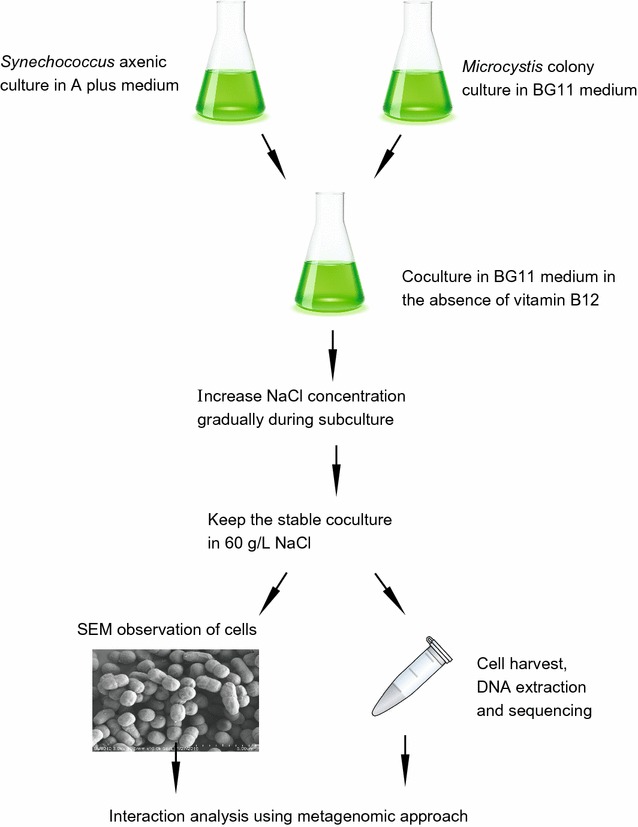
The flow diagram shows the establishment of the coculture system in the study

**Fig. 2:**
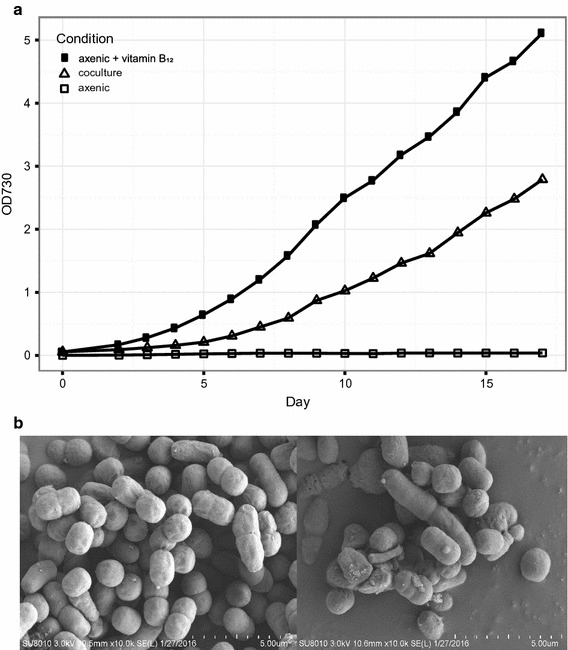
The comparison of algae cell in coculture and axenic culture. **a** The growth curve of algae cell in coculture (open triangles), axenic *Synechococcus* sp. PCC 7002 in the presence (filled squares) or absence (open squares) of vitamin B_12_. The basal medium of the cultures is BG11 medium (see “Materials and methods” section). Obviously, the axenic cyanobacterium is unable to grow in the absence of vitamin B_12_; **b** the scanning electron microscopy (SEM) image of cells in coculture (bottom right panel) and axenic culture (bottom left panel)

At the beginning, our coculture was established by mixing the xenic *Microcystis* colony and pure *Synechococcus* sp. PCC 7002 strain in the BG 11 medium (Fig. 1). During the step-wise increase of the salinity in the medium (see “Materials and methods” section for the details), *Microcystis aeruginosa* strain is believed to be absent in the coculture, which is confirmed that the amplification of the conserved marker genes in *Microcystis* (Additional file 1: Figure S1, Table S1). Our observation is consistent with the previous experimental result that *Microcystis aeruginosa* PCC 7806 endured temporary salinity as high as 17.5 g l^−1^ and was unable to maintain the growth under high salinity (Tonk et al. 2007). After the coculture was established stably, the growth of the cells in three condition was compared (Fig. 2a). The strain *S.* sp. PCC 7002 is unable to grow in the axenic culture in the absence of vitamin B_12_, but grow in the presence of vitamin B_12_ as expected, confirming the previous observation that the cyanobacterium required vitamin B_12_ for growth in the laboratory (Wilhelm and Trick 1995). In the first 2 days after subculture, there is no significant difference in growth rate between coculture and axenic culture supplemented with B_12_ (Fig. 2a). After that, the cells in the stable coculture grew slower than cells in the axenic culture. On the 15th day after subculture, the cell density in the axenic culture was almost twice that in the coculture. Based on the OD values, the doubling time of two cultures under high salinity is estimated, with ~ 38 h in the axenic culture and ~ 45 h in the coculture. The significant difference in growth rate of cells between two conditions could be explained by several possible factors, including insufficient cobalamin, shortage of nutrients, or high salinity in the coculture. In the coculture where *S.* sp. PCC 7002 can maintain its growth, there may exists vitamin B_12_ to support the growth of *S.* sp. PCC 7002 and the possible cobalamin producer may be some bacteria in the coculture. Furthermore, the scanning electron microscopy (SEM) imaging of the coculture showed that cells with multiple morphological characteristics, including oval and long rod shapes, coexist in the coculture, whereas only an oval morphology was observed in the axenic sample (Fig. 2b). The coccus shape observed in the coculture was consistent with the shape of *S.* sp. PCC 7002 in the axenic culture, indicating that there was more than one species present in the coculture.

### The composition of the coculture revealed through metagenomics analysis

To investigate the composition and possible cobalamin producer in the coculture, metagenomics approach was applied in our study. The raw sequence was filtered (Additional file 1: Table S2**)** and then assembled using three metagenome assembly software (Additional file 1: Table S3). Among the assembly, the result produced by SPAdes software (Bankevich et al. 2012) was selected and subject to the following analysis. Three genome bins were reconstructed from the assembly of the coculture sample (Table 1; Additional file 1: Table S4). And the number of genome bin agreed with the number of 16S rRNA genes predicted from the final assembly. Two of three genome bins were 100 and 99% complete, respectively, based on using marker genes specific to a genome’s inferred lineage within the reference genome tree (Parks et al. 2015), indicating that the approach of deep metagenomic sequencing for a simple coculture could improve the quality of assembled genome sequence for the individual members in the coculture.

**Table 1:** Statistics of each genome bin reconstructed from coculture

Group	Total size (bp)	No. scaffold	Longestcontig (bp)	N50 (bp)	GC content (%)	Completeness (%)^a^	Contamination (%)^a^	Relative abundance (%)^b^	Species name
Group1	4,830,778	38	781,449	506,457	63.63	99.8/98.28	1.5/0	6.68	*Pseudomonas stutzeri* TAIHU
Group2	4,926,263	18	1,392,957	1,200,254	63.43	99.51/100	1.48/0	9.19	*Mesorhizobium* sp. TAIHU
Group3	3,401,077	39	976,673	479,380	49.2	100/100	0/0	84.14	*Synechococcus* sp. PCC7002

^a^The completeness and contaimnation were determined by the CheckM software, the first value before the slash was the result of lineage-specific workfow and the latter one was the result of taxonomy-specific workflow in the program

^b^The relative abundance was the proportion of reads on each genome bin in the total merged read, excluding paired read and the alignment was performed by the BWA software

**Fig. 3:**
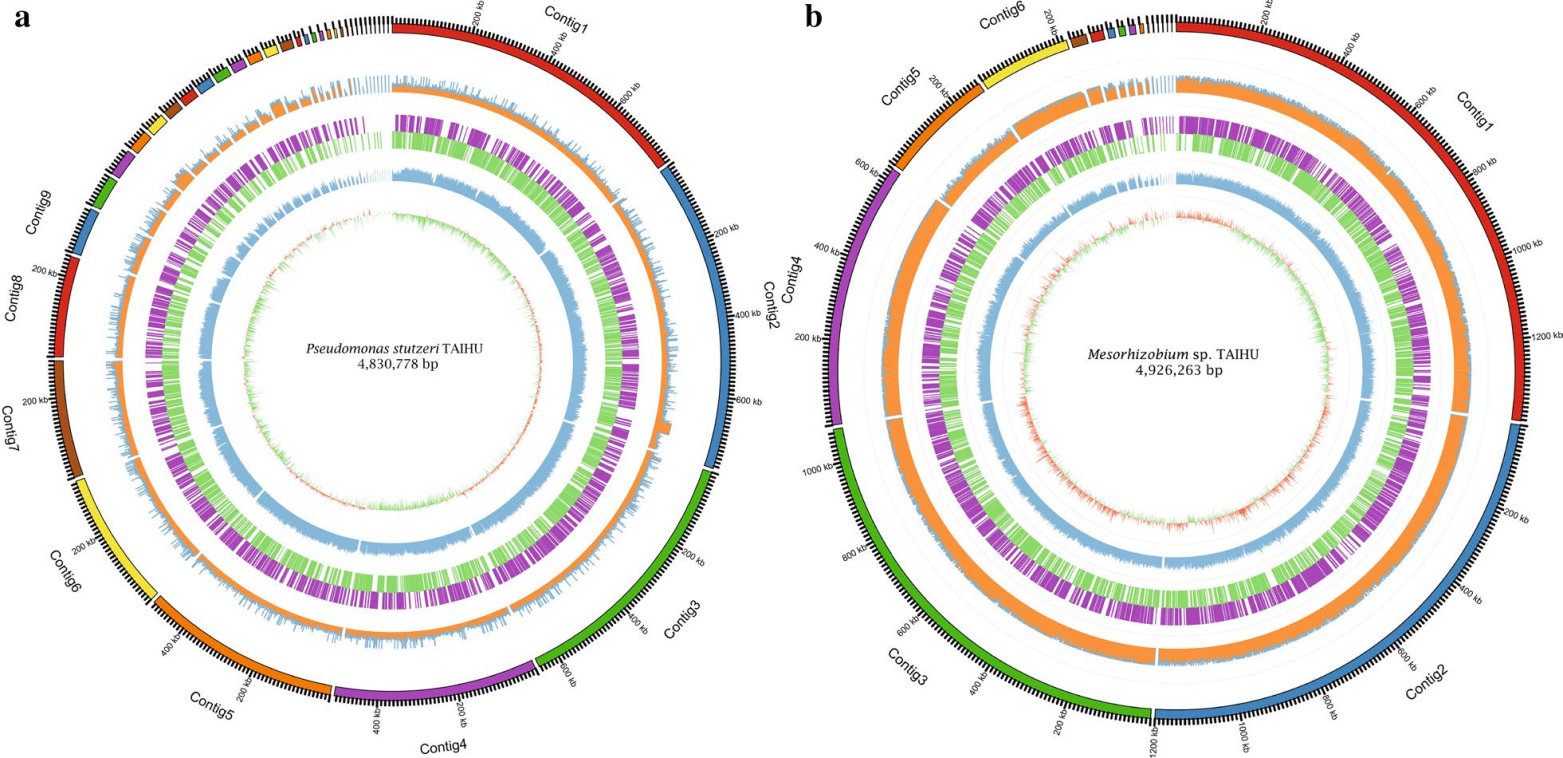
The representation of two bacteria genome sequence recovered from the coculture. The genomic characterization of *Mesorhizobium* sp. TAIHU (**a**) and *Pseudomonas stutzeri* TAIHU (**b**) is illustrated in the graphic representation generated by Circos program. The rings from outer to inner indicate the contigs, read coverage of each contigs (orange, 1 Kbp window), gene distribution on each strand (positive: purple, negative: green), GC content (blue, 1 Kbp window) and GC skew (orange above and green below zero, 1 Kbp window)

Based on the binning result, our coculture contained other two bacteria besides *Synechococcus* sp. PCC 7002. The sequence similarity of the 16S rRNA genes showed that one bacterium was closely related to *Pseudomonas stutzeri* strains and the other affiliated with the *Mesorhizobium* genus. In a phylogenetic tree constructed from the complete 16S rRNA sequences of all *P. stutzeri* strains (Additional file 1: Figure S2), the strain in the coculture was clustered with *P. stutzeri* T13, an aerobic denitrifying bacterium isolated from activated sludge of a wastewater plant (Li et al. 2012). The estimated DNA–DNA hybridization (DDH) values (Meier-Kolthoff et al. 2013) between eight *P. stutzeri* strains (including *P. stutzeri* T13) and the strain in the coculture were greater than 79% (Additional file 1: Table S5), and the corresponding probability that these strains belonged to the same species is higher than 90%. Furthermore, the average nucleotide identity (ANI) values between the eight *P. stutzeri* strains and the strain identified here ranged from 97.88 to 98.57% (Additional file 1: Table S5), higher than the threshold of 96% proposed by Richter and Rossello-Mora (2009) to circumscribe the prokaryotic species. Taken together, the strain in our coculture was designated *Pseudomonas stutzeri* TAIHU (Fig. 3). The same analysis strategy was utilized to delineate another bacterium in the coculture (Additional file 1: Table S6), referred to as *Mesorhizobium* sp. TAIHU (Fig. 3). The bacterium was clustered with *M.* sp. UASWS1009 in the phylogenetic tree of 16S rRNA in the *Mesorhizobium* genus (Additional file 1: Figure S3); the latter is a nitrifying bacterium collected from sewage sludge (http://www.ncbi.nlm.nih.gov/nuccore/758551542).

### Metabolism of vitamin B_12_ in the coculture: biosynthesis and transport

**Fig. 4:**
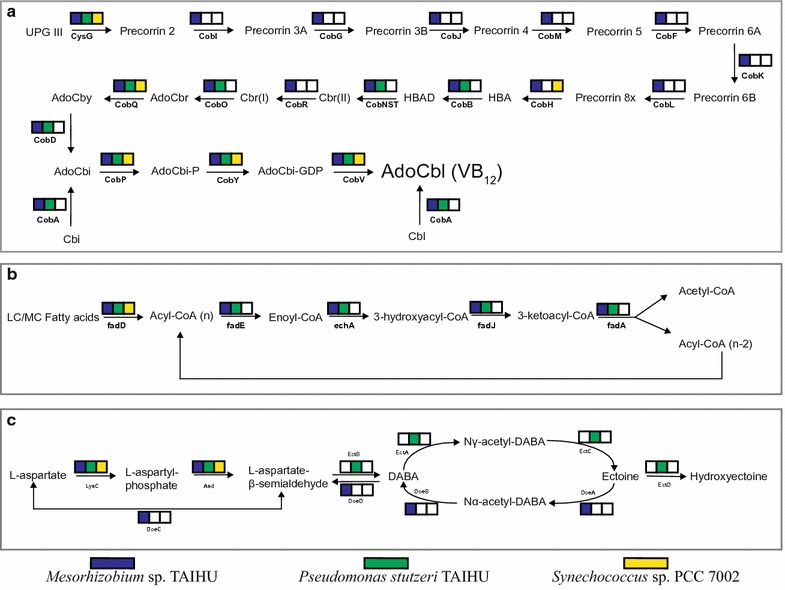
The metabolic pathway analysis for three members in the coculture. **a** The aerobic cobalamin biosynthesis pathway; **b** the β-oxidation pathway; **c** the ectoine biosynthesis and degration pathway. The color box indicates that the individual in the coculture carries the gene encoding the enzyme involved in the pathway, whereas an empty box (white) indicates the absence of the corresponding gene. The genes in *Synechococcus* sp. PCC 7002, *Mesorhizobium* sp. TAIHU and *Pseudomonas stutzeri* TAIHU are shown in yellow, blue and green, respectively. The abbreviations of the metabolites and enzymes used in the figure refer to the reference pathway (map00860, map00071, map00260) in KEGG database and the paper by Swither et al. (2012)

*Synechococcus* sp. PCC 7002 was reported to require vitamin B_12_ (cobalamin) for in vitro growth (Wilhelm and Trick 1995) and it was confirmed in our study (Fig. 2). Through the genome analysis, most of the genes involved in the cobalamin biosynthesis were found to be absent in the genome of *S.* sp. PCC 7002 (Fig. 4a), that explains the inability of this organism to synthesize cobalamin de novo. The strain *S.* sp. PCC 7002 grew well in the coculture in the absence of cobalamin, suggesting that one or both of the other two bacteria in the coculture have the capacity to synthesize vitamin B_12_. The annotation results for the genomes of two heterotrophic bacteria support this hypothesis. The bacterium *Mesorhizobium* sp. TAIHU carries all genes involved in the aerobic pathway of cobalamin biosynthesis (Fig. 4a). These genes are distributed into five clusters (Additional file 1: Figure S4), which are located on two contigs. The largest cluster contains 15 adjacent genes. The genes encoding an aerobic cobalt chelatase composed of CobN, CobS and CobT subunits, was present in the genome of *M.* sp. TAIHU. The ATP-dependent chelatase is responsible for the insertion of cobalt ions in the late stage of the aerobic cobalamin biosynthesis (Rodionov et al. 2003). As in the genome of *M. loti* MAFF303099 (Kaneko et al. 2000), the genes encoding *cobS* and *cobT* subunits are clustered together and separated from other cobalamin genes in the genome of *M.* sp. TAIHU (Additional file 1: Figure S4). The annotation of the *Pseudomonas stutzeri* TAIHU genome shows that it lacks most of the genes involved in the synthesis of the corrin ring structure and the gene (*cobR*) encoding cobalt reductase (Fig. 4a), showing its incapability of synthesizing cobalamin de novo. However, *P. stutzeri* TAIHU contains the gene encoding cob(I)alamin adenosyltransferase (*cobA*), which is responsible for the synthesis of adenosylcobalamin (coenzyme vitamin B_12_) from cobalamin and the synthesis of adenosylcobinamide from cobinamide as a salvage pathway (Escalante-Semerena 2007; Swithers et al. 2012). And this potential enables the bacterium to save the great deal of energy spending on the biosynthesis of the corrin ring structure (Escalante-Semerena 2007).

Genes responsible for the transport of cobalt and cobalamin were also investigated in members of the coculture. The gene *btuB* was identified in the genome of *Mesorhizobium* sp. TAIHU (Table 2). The outer membrane receptor BtuB is responsible for the transport of vitamin B_12_ and other corrinoids in prokaryotic cells (Rodionov et al. 2003). The genes for the complete vitamin B_12_ ABC transporter, consisting of ATPase component BtuD, permease component BtuC and B_12_-binding component BtuF, were found adjacent to the BtuB receptor in *M.* sp. TAIHU. The arrangement was consistent with previous reports that the B_12_-specific components of transporters in Gram-negative bacteria are BtuBFCD (Rodionov et al. 2003; Zhang et al. 2009). The BtuB receptor was also identified in *Pseudomonas stutzeri* TAIHU, and was encoded next to the gene for the B_12_-binding component BtuF of the ABC transporter (Table 2). Although the cobalamin transport genes were not identified in the genome of *S.* sp. PCC 7002 based on the annotation from RAST (Aziz et al. 2008) and Prokka (Seemann 2014), a recent study by Bryant and colleagues discovered the genes responsible for the cobalamin transport (*btuBCF*) in the cyanobacterium, which were previously annotated as siderophore uptake genes (Pérez et al. 2016a, b). In addition, the role of these genes in the cobalamin transport was demonstrated experimentally in their study (Pérez et al. 2016a, b).

**Table 2:** Statistics of functional genes of the three species

Function pathway or enzymes	*Synechococcus* sp. PCC 7002^a^	*Mesorhizobium* sp. TAIHU	*Pseudomonas stutzeri* TAIHU
Energy source and carbon source			
Photosynthesis and CO_2_ fixation	√		
Beta-oxidation of fatty acid		√	√
Cobalamin synthesis and transport			
Cobalamin de novo synthesis		√	
Cobalamin salvage		MSTH_00675	PSTH_00549/01543
Cobalamin and corrinoids transport (*btuBFCD*)	SYNPCC7002_A0634/0635/0637	MSTH_03067-03070	
Cobalamin-associated enzymes			
Cobalamin-dependent methionine synthase (*metH*)	SYNPCC7002_A2466	MSTH_00354/00815	PSTH_03850
Cobalamin-independent methionine synthase (*metE*)		MSTH_01286	PSTH_01866
Methylmalonyl-CoA mutase		MSTH_01813/02120	
Cobalamin-dependent ribonucleotide reductase (class II)		MSTH_00664	PSTH_00904 PSTH_00905
Cobalamin-independent ribonucleotide reductase (class I)	SYNPCC7002_A1350 SYNPCC7002_A0382		PSTH_02138 PSTH_02139
Anaerobic ribonucleoside-triphosphate reductase (RNR class III)			PSTH_03057 PSTH_03058
Compatible solutes			
Ectoine synthesis (*ectABCD*)			PSTH_01019-01022
Glycine betaine (*betAB*)		MSTH_02104/02105/02862	PSTH_02582/02583
Trehalose synthesis (*ostAB*)		MSTH_01326/01325	PSTH_04112/04113
Trehalose (*treY, treZ*)			PSTH_03770/03772
Trehalose (*treS*)			PSTH_03785
Sucrose (*sppA, spsA*)	SYNPCC7002_A0887/0888		
Glucosylglycer-(ol/ate) (*ggpS, ggpP*)	SYNPCC7002_A2841/2851/2852		
Transport of glycine betaine/proline (*proVWX*)		MSTH_01321-01323 MSTH_03779-03781	PSTH_04547-04549
Ectoine degradation (*doeABCD*)		MSTH_03098/03099 MSTH_03109/03110	

^a^The check mark indicates that the organism contains the genes responsible for the metabolic ability dictated in the column of the table. Among these, beta-oxidation of fatty acid and biosynthesis of cobalamin are shown in Fig. 4

### Biosynthesis of compatible solutes in the coculture

Besides the absence of cobalamin, the medium where the coculture grows is supplemented with 1 M NaCl. Obviously, the high salinity of the medium would impact the growth of the cells in the coculture (Fig. 2a), although the coculture can maintain its growth stably in the laboratory. To maintain normal metabolic activities under hyperosmotic stress, the production or uptake of compatible solutes (e.g., glycine betaine, sucrose, trehalose and ectoine) is one of strategies that the prokaryotes used (Empadinhas and da Costa 2008; Hagemann 2011; Klahn and Hagemann 2011). On the basis of pathway analysis and comparison, genes involved in the biosynthesis of different compatible solutes in each member were identified to mediate the high osmotic stress in the extracellular environment.

In *S.* sp. PCC 7002, experiment evidence showed that sucrose and glucosylglycerol (GG) were detected as the major compatible solutes (Klaehn et al. 2010). Moreover, glucosylglycerate (GGA) as a minor compatible solute was also observed to accumulate in the cyanobacterium. The genes responsible for the biosynthesis of these compounds was identified in the genome. The biosynthesis of GG and GGA was catalyzed by multiple enzymes, including glucosylglycerol-phosphate synthase (GgpS), glucosylglycerol 3-phosphatase (GgpP), and glycerol-3-phosphate dehydrogenase (Ludwig and Bryant 2012). The sucrose synthesis was encoded by two genes in *S.* sp. PCC 7002 (*sppA* and *spsA*) (Cumino et al. 2010). In contrast, the genes for the synthesis of sucrose, GG and GGA was found absent in the two heterotrophs (Table 2). In the case of *P. stutzeri* TAIHU, the operon (*ectABCD*) consisting of four ORFs for the biosynthesis of ectoine was identified. Ectoine, also known as tetrahydropyrimidine ectoine, and its derivative 5-hydroxyectoine, are widely used as compatible solutes among microbial communities (Galinski et al. 1985; Cánovas et al. 1998; Pastor et al. 2010). In addition, the other two common compatible solutes glycine betaine (GB) and trehalose were possibly produced by both heterotrophic bacteria, since the genes for biosynthesis pathway (*betAB* and *ostAB*) were found in their genomes. Among five pathways for trehalose synthesis (Paul et al. 2008; Moussaid et al. 2015), the *ostAB* pathway shared in two heterotrophs is most widespread in eubacteria, archaea and plants. The *P. stutzeri* TAIHU also harbor the genes involved in the two pathway for synthesizing trehalose, that is TreY-TreZ pathway and TreS pathway (Paul et al. 2008). Although the accumulation of trehalose and GB was demonstrated in many cyanobacteria (Hagemann 2011), the genes associated with these osmolytes synthesis was not identified in *S.* sp. PCC7002 in our analysis. The previous evidence that GB was not detected in the axenic culture of *S.* sp. PCC 7002 with comparable salinity further corroborated our observation (Cumino et al. 2010).

### Potential interaction in the coculture

In the coculture established here, the vitamin-supplying heterotrophs must have an obligate dependency on the cyanobacterium *S.* sp. PCC 7002 for the fixed carbon. As essential components of membranes, fatty acids are important sources of carbon and energy in all living organisms. Once uptake by bacteria, fatty acids can be degraded via the β-oxidation pathway which include the conversion of acyl-CoA to enoyl-CoA, hydration, oxidation and thiolysis, resulting in the acyl-CoA minus two carbons and acetyl-CoA (Fujita et al. 2007). Through the metabolism analysis for three members in coculture, the genes encoding for the enzymes involved in the degradation pathway were present in the two heterotrophic bacteria whereas absent in the cyanobacterium (Fig. 4b). The gene presence/absence pattern in the fatty acid degradation pathway are suggestive of a possibility that the fatty acid generated by the cyanobacterium might supply the carbon and energy source for the heterotrophies in the coculture.

It is reported that the compatible solutes not only play an important role in protecting against the hyperosmotic stress, but also represent a significant proportion of total cell carbon and/or nitrogen (Welsh 2000; Vargas et al. 2006). As described above, carbohydrate compatible solutes generated by the cyanobacterium *S.* sp. PCC 7002, such as sucrose and GG can be exploited as potential carbon and energy source by the heterotrophies in coculture. And we have found that two heterotrophic bacteria harbor the genes involved in the phosphoenolpyruvate phosphotransferase system (PTS), with fructose-specific component in the case of *P. stutzeri* TAIHU and mannose-specific component in the case of *M.* sp. TAIHU. The PTS is reported to participates the transport and phosphorylation of specific carbohydrates in bacteria, including saccharides, amino sugars and other sugar derivatives (Deutscher et al. 2006). Another interesting observation is that, although the genes for ectoine biosynthesis were identified in *P. stutzeri* TAIHU as described earlier, the operon responsible for the degradation of ectoines (*doeABCD*) was found absent in *P. stutzeri* TAIHU, but present exclusively in *M.* sp. TAIHU (Fig. 4c). The ectoine degradation is catabolized through four-step reactions (Fig. 4c), supplying aspartate as final product for the cell (Table 2). Moreover, *M.* sp. TAIHU carries two copies of the operons for one high-affinity transporter system for glycine betaine (*proXWV*). Early evidences showed that the uptake of ectoine depend on the glycine betaine transport system in *Escherichia coli* (Jebbar et al. 1992) and a halophilic bacterium, *Chromohalobacter salexigens* (Vargas et al. 2006). These observations allow us to infer that *M.* sp. TAIHU might utilize the ectoine secreted by *P. stutzeri* TAIHU, indicating a potential interaction between both heterotrophic bacteria in the community.

## Discussion

Due to the inability to tolerate the high salinity (Tonk et al. 2007), the freshwater cyanobacterium *Microcystis* species in the colony was replaced by the euryhaline cyanobacterium *S.* sp. PCC 7002, resulting in the establishment of the stable coculture system, which is characterized by the high salinity (~ 1 M NaCl) and maintained for 5 years in the laboratory. The replacement of photoautotroph in the micro-community has the following advantages: (i) *S.* sp. PCC 7002 is naturally transformable, making easier molecular genetic operation (Stevens and Porter 1980; Frigaard et al. 2004); (ii) The doubling time for the species is comparably short under optimal conditions (Ludwig and Bryant 2012); Based on these superior characteristics, the coculture system established here provides a useful model to study the microbial interaction between cyanobacteria and bacteria, and the mechanisms for mediating the high salinity in a cooperated way. In addition, further understanding of the nature of interaction will benefit from the genetic transformation of *S.* sp. PCC 7002, the only photoautotroph in the coculture system.

In our coculture system, both bacteria have the metabolic potential to refresh the cobalamin through biosynthetic or salvage pathway, and that is required by the B_12_ auxotroph *S.* sp. PCC 7002. Vitamin B_12_ are essential cofactor for several important enzymes and methionine synthase is one of most prominent one (Rodionov et al. 2003). The biosynthesis of methionine in organisms can be catalyzed by two isozymes of methionine synthase, namely the vitamin B_12_-dependent one (MetH) and B_12_-independent one (MetE). The MetH, also known as 5-methyltetrahydrofolate-homocysteine methyltransferase (N5-MeTHF), catalyzes methyl group transfer from methyltetrahydrofolate to homocysteine to create methionine using B_12_ as a cofactor (Zydowsky et al. 1986). The cyanobacterium *S.* sp. PCC 7002 only contains the B_12_-dependent methionine synthase gene (*metH*) yet cannot synthesize B_12_ (Fig. 4a), giving a possible explanation that the strain requires exogenous vitamin B_12_ for growth in the laboratory (Fig. 2a). And a recent molecular study on *S.* sp. PCC 7002 have demonstrated the hypothesis and proposed that the enzyme MetH is probably the only enzyme in the cyanobacterium that requires cobalamin as cofactor (Pérez et al. 2016a). In their study, *S.* sp. PCC 7002 was shown to maintain growth in the absence of cobalamin with added methionine, although less than the growth rate under normal condition (with cobalamin). Based on these evidences, we cannot rule out another possible scenario that the heterotrophic bacteria in the coculture supply the methionine and its derivatives, not just cobalamin, for the cyanobacterium (Xie et al. 2016). In either case, *S.* sp. PCC 7002 always benefit from the bacteria in the coculture. It should be pointed out that the biosynthesis of cobalamin in the coculture system is not only important for the cyanobacterium, but also for the cobalamin-producer *M.* sp. TAIHU. Genes encoding the B12-requiring enzymes, including the methylmalonyl-CoA mutase (MCM) and ribonucleotide reductase (RNR class II) were identified in the genome of *M.* sp. TAIHU (Table 2).

Based on the genomic analysis, each member in the coculture system is found to harbor the genes involved in the biosynthesis of specific compatible solute to cope with the hyperosmotic stress. In addition to stress protection, these diverse compatible solutes also represent a significant proportion of cell carbon and/or nitrogen source, which may be exploited by the community as carbon and energy source, once released to the environment (Welsh 2000; Vargas et al. 2006). Since the medium used for the coculture isn’t supplemented with any organic carbon source, the photosynthates generated from the cyanobacterium *S.* sp. PCC 7002, including compatible solutes, is considered to be the final source of fixed carbon and energy for the whole community. For example, the carbohydrate compatible solutes (sucrose, GG and GGA) produced by *S.* sp. PCC 7002 may be utilized by the heterotrophic bacteria. Moreover, both heterotrophic bacteria harbor the genes involved in the phosphoenolpyruvate phosphotransferase system (PTS), which is associated with the transport and phosphorylation of specific carbohydrates in bacteria (Deutscher et al. 2006). Alternatively, the bacteria may obtain the carbon and energy source through beta-oxidation of fatty acid since the genes involved in the catabolism of fatty acid are exclusively present in the heterotrophic bacteria but absent in the cyanobacterium (Fig. 4b).

The approach of establishing an artificial system combined with deep sequencing metagenomics, represents a novel attempt to investigate in greater depth the interesting microbial components in communities. The similar mutualism based on cobalamin and nutrient exchange have been described in *Microcystis* colony (Xie et al. 2016), green algae (Croft et al. 2005; Kazamia et al. 2012), microbial mat consortia (Cole et al. 2014; Romine et al. 2017), and diatom (Durham et al. 2015). Although the coculture system cannot represent the entire microbial diversity in a natural community, the insight gained from the analysis of the simpler coculture could help to develop a fundamental understanding of the interaction between complex community in nature. Given the advantage of natural genetic transformation for *S.* sp. PCC 7002 (Frigaard et al. 2004), the stable coculture system established here provides a useful model to decipher the nature of interaction between cyanobacteria and heterotrophic bacteria.

## Additional file

**Additional file 1.** Additional figures and tables.

## Abbreviations

ANI: average nucleotide identity
DDH: DNA–DNA hybridization
SEM: scanning electron microscopy
PTS: phosphoenolpyruvate phosphotransferase system
OD: optical density
