# Supplementary material for: Metagenomic analysis reveals potential interactions in an artificial coculture
Source: AMB Express. 2017 Nov 2;7:193. doi: 10.1186/s13568-017-0490-2 (PMC5668215; doi:10.1186/s13568-017-0490-2)
Supplement: Supplementary file 1 — Additional file 1. Additional figures and tables. [file 13568_2017_490_MOESM1_ESM.docx]

AMB Express: Additional Material

**Metagenomic analysis reveals potential symbiotic interactions in an artificial coculture**

Minglei Ren^1, 3, 5^, Guiying Zhang^1, 4, 5^, Zi Ye^1^, Zhixian Qiao^1^, Meili Xie^1^, Yan Lin^1^, Tao Li^1*^, Jindong Zhao^1, 2^

^1^State Key Laboratory of Freshwater Ecology and Biotechnology, Institute of Hydrobiology, Chinese Academy of Sciences, Wuhan 430072, China

^2^College of Life Science, Peking University, Beijing 100871, China

^3^Present address: Simon F. S. Li Marine Science Laboratory, School of Life Sciences, The Chinese University of Hong Kong, Shatin, Hong Kong SAR, China

^4^Present address: Southern University of Science and Technology, Shenzhen 518055, China

^5^These authors contributed equally to this work

**^*^Correspondence:**

Tao Li, Key Laboratory of Algal Biology, Institute of Hydrobiology, Chinese Academy of Sciences, Wuhan 430072, China, Email: [litao@ihb.ac.cn](mailto:litao@ihb.ac.cn), Tel: +86 136 6722 2098.

**Total pages: 16; Total number of figures: 4; Total number of tables: 7.**

**Figures**

**Fig. S1** The PCR amplification result of three marker genes in the cells from three cultures. The name of genes is shown above the gel electrophoresis image, and different cultures are shown below the image. Among the culture, “axenic 7002” and “axenic 98” represent the cells of axenic *Synechococcus* sp*.* PCC 7002 and axenic *Microcystis* aeruginosa TAIHU98, respectively. The sequence of the primers used in the PCR amplification of marker genes is indicated in Table S1.

**Fig. S2** Phylogenetic location of *Pseudomonas stutzeri* TAIHU in all strain of *P. stutzeri* with genome sequence available. Neighbor-Joining tree were constructed with full 16S RNA nucleotide sequence from 23 strains belonging to *P. stutzeri* using MEGA6 software. Bootstrap values are listed at the node, expressed as percentages of 500 replications. The scale bar represents 0.8 changes per nucleotide position The *P. stutzeri* TAIHU strain is marked in red color.

**Fig. S3** Phylogenetic location of *Mesorhizobium sp.* TAIHU in all strains of *Mesorhizobium* genus with genome sequence available. Neighbor-Joining tree were constructed with full 16S RNA nucleotide sequence from 81 genomes belonging to *Mesorhizobium* genus using MEGA6 software. Bootstrap values are listed at the node, expressed as percentages of 500 replications. The scale bar represents 3 changes per nucleotide position. The *M. sp.* TAIHU strain is marked in red color.

**Fig. S4** The location of genes involved in the aerobic cobalamin biosynthesis on both bacterial genomes. The distribution of all genes associated with cobalamin biosynthesis in the genomes of *Mesorhizobium sp.* TAIHU (a) and *Pseudomonas stutzeri* TAIHU (b). The gene ID of each genome is based on the annotation of Prokka software. The scale bars are 10 Kbp (a) and 5 Kbp (b), respectively.

**Tables**

**Table S1.** The primers used in the PCR amplification of marker genes in the coculture and axenic culture (see Figure S1).

**Table S2.** Statistics of the clean reads from the coculture sample in this study.

**Table S3.** Comparison of the assembly of using different assembly software.

**Table S4.** Statistics of annotation results for two heterotrophic bacteria in coculture.

**Table S5.** The average nucleotide identity (ANI) value and estimated DNA-DNA hybridization (DDH) values between the two bacteria in coculture and other known strains in *Pseudomonas stutzeri*.

**Table S6.** The average nucleotide identity (ANI) value and estimated DNA-DNA hybridization (DDH) values between the two bacteria in coculture and other known strains in *Mesorhizobium* genus.

**Table S7.** The scaffold lists of two heterotrophic bacteria in coculture sample.


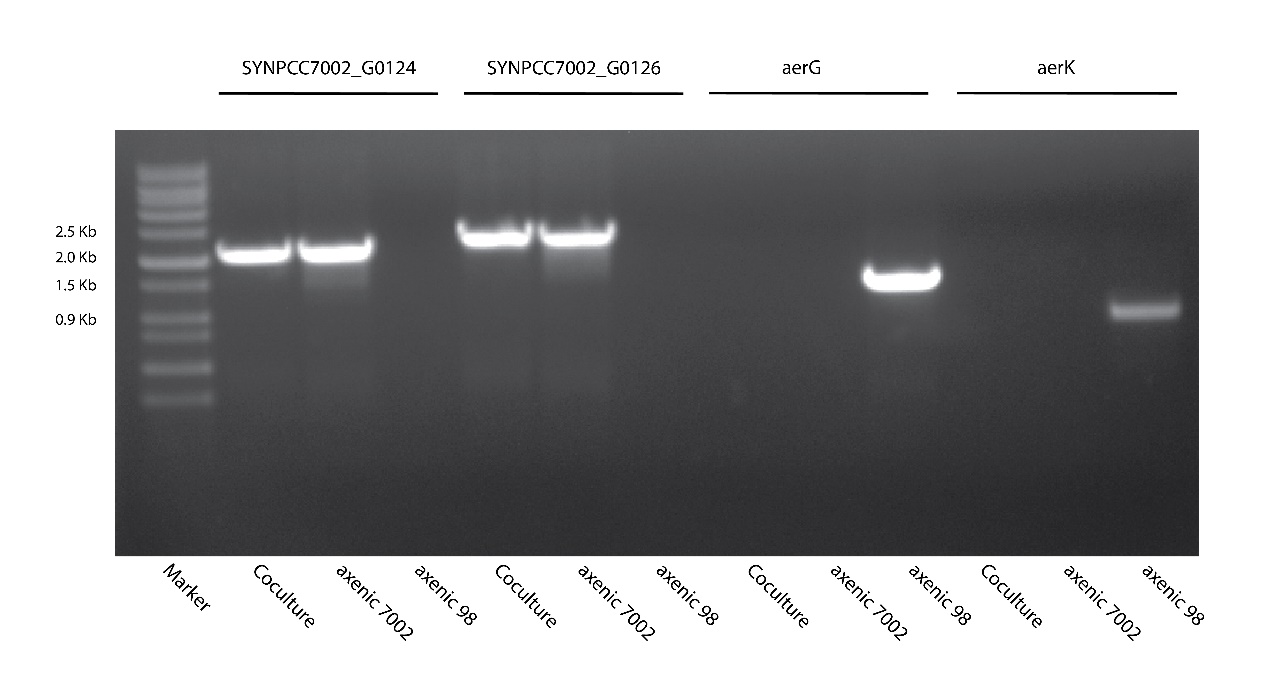


**Fig. S1** The PCR amplification result of three marker genes in the cells from three cultures. The name of genes is shown above the gel electrophoresis image, and different cultures are shown below the image. Among the culture, “axenic 7002” and “axenic 98” represent the cells of axenic *Synechococcus* sp*.* PCC 7002 and axenic *Microcystis* aeruginosa TAIHU98, respectively.


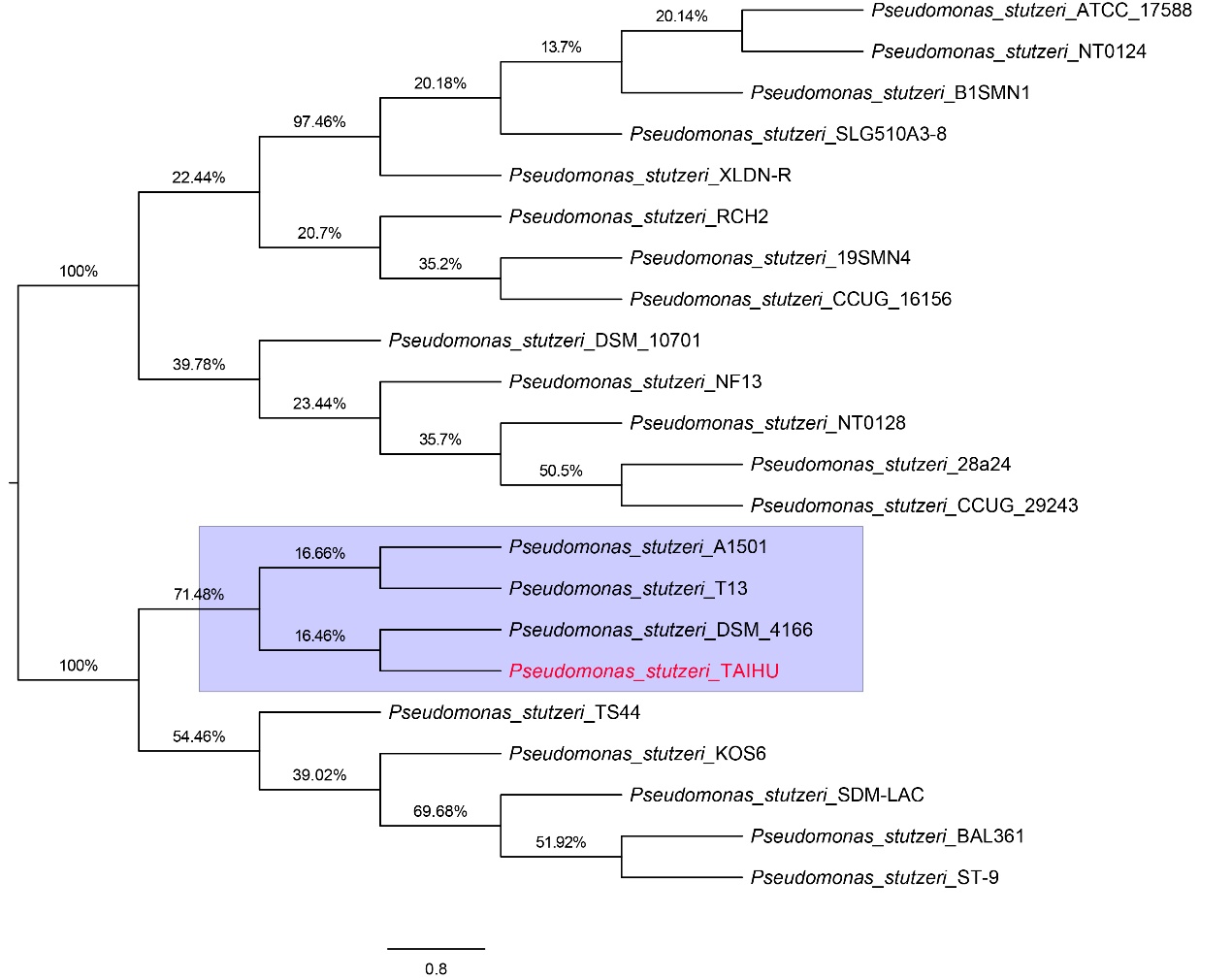


**Fig. S2:** Phylogenetic location of *Pseudomonas stutzeri* TAIHU in all strain of *Pseudomonas stutzeri* with genome sequence available. Neighbor-Joining tree were constructed with full 16S RNA nucleotide sequence from 23 strains belonging to *Pseudomonas stutzeri* using MEGA6 software. Bootstrap values are listed at the node, expressed as percentages of 500 replications. The scale bar represents 0.8 changes per nucleotide position The *Pseudomonas stutzeri* TAIHU strain is marked in red color.


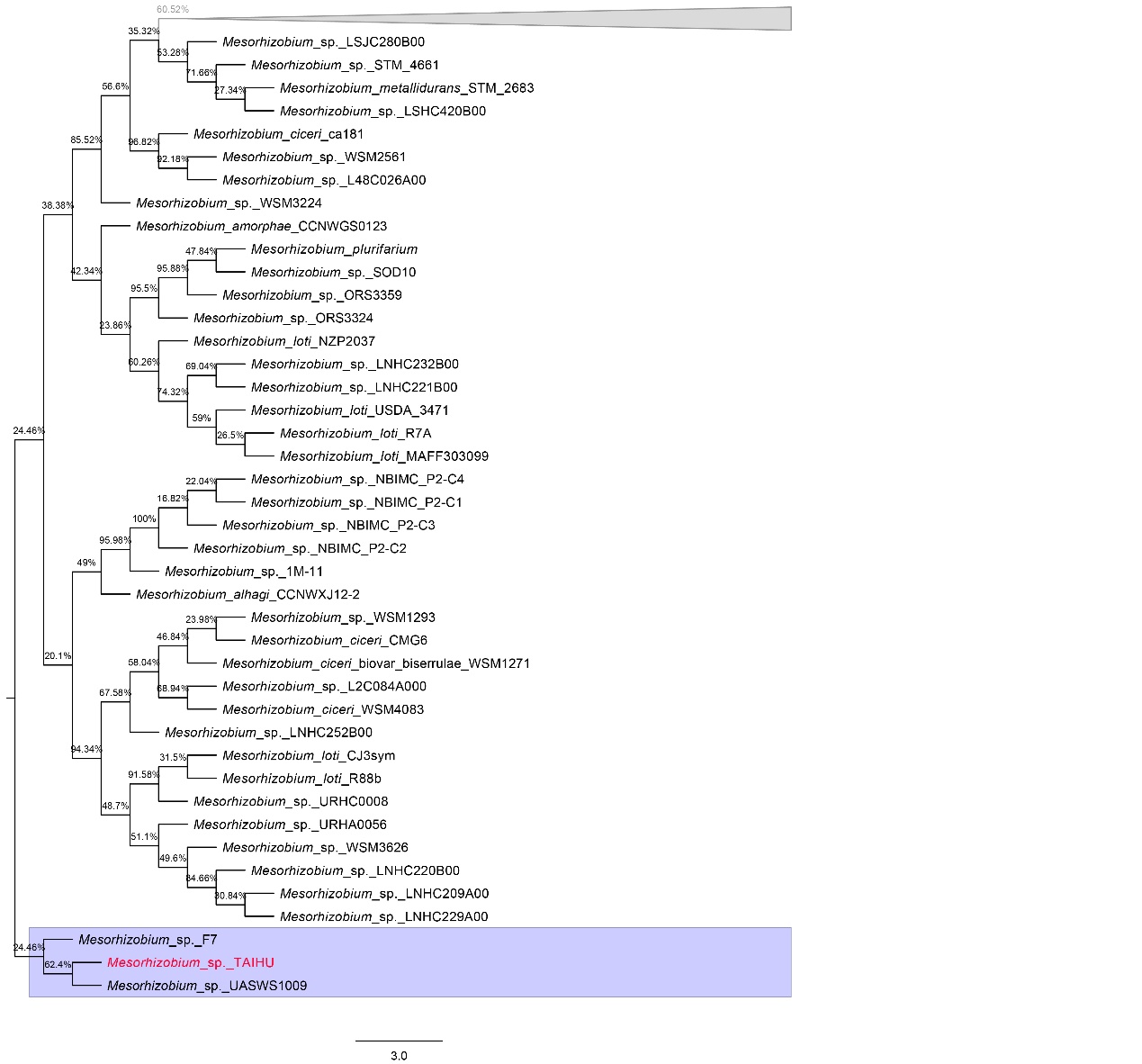


**Fig. S3:** Phylogenetic location of *Mesorhizobium sp.* TAIHU in all strains of *Mesorhizobium* genus with genome sequence available. Neighbor-Joining tree were constructed with full 16S RNA nucleotide sequence from 81 genomes belonging to *Mesorhizobium* genus using MEGA6 software. Bootstrap values are listed at the node, expressed as percentages of 500 replications. The scale bar represents 3 changes per nucleotide position. The *Mesorhizobium sp.* TAIHU strain is marked in red color.


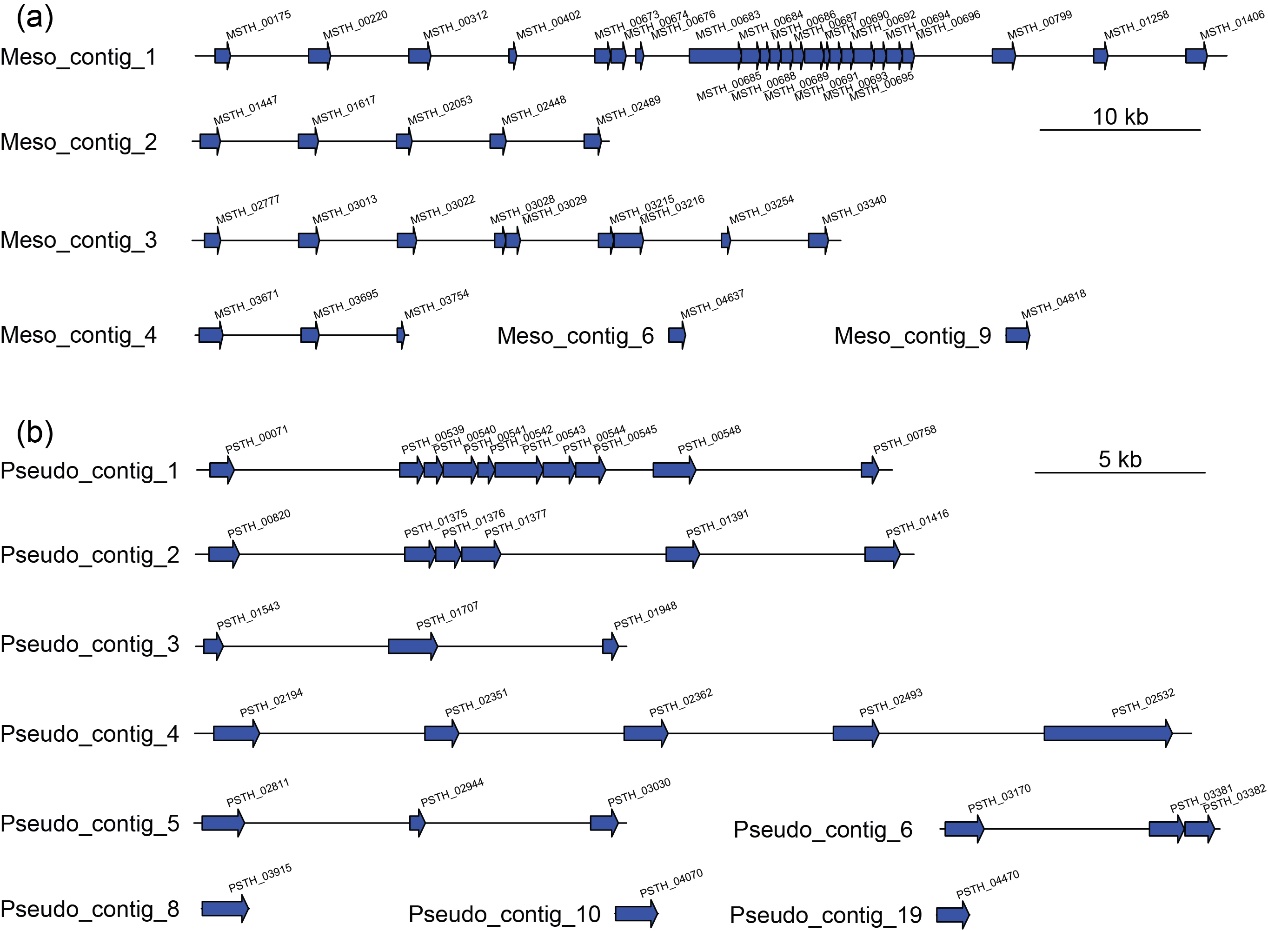


**Fig. S4: ­**The location of genes involved in the aerobic cobalamin biosynthesis on both bacterial genomes. The distribution of all genes associated with cobalamin biosynthesis in the genomes of *Mesorhizobium sp.* TAIHU (A) and *Pseudomonas stutzeri* TAIHU (B). The gene ID of each genome is based on the annotation of Prokka software. The scale bars are 10 Kbp (A) and 5 Kbp (B), respectively.

**Table S1**

The primers used in the PCR amplification of marker genes in the coculture and axenic culture used in this study.

| Oligonucleotide | Oligonucleotide Sequence (5’-> 3’) |
| --- | --- |
| SYNPCC7002_G0124_F | AACAAGCTTATTTCAACTGGG |
| SYNPCC7002_G0124_R | TCCTTTTGCTGACTAAACCAC |
| SYNPCC7002_G0126_F | GAAAACAGCCAGGCATCGAAG |
| SYNPCC7002_G0126_R | ATGTTTCTGATAATGTGCCCAA |
| aerG_F | TACCTTACCCGTTCGCATC |
| aerG_R | CTGGTAACGATATCCGCTTC |
| aerK_F | AAAAGTGAACATCTGCGAGT |
| aerK_R | TCTTCTGATTCAATCGCTGT |

**Table S2.**

Statistics of the clean reads from the coculture sample in this study.

|  | **Coculture sample** | **Num_of_reads** | **Read_length**  **(bp)** | **Total_base**  **(Mb)** |
| --- | --- | --- | --- | --- |
| 1st sequencing | paired-end reads | 1,350,598*2 | 50-301 | 480 |
|  | merged & single reads | 7,389,347 | 50-559 | 1705 |
| 2nd sequencing | paired-end reads | 1,481,448*2 | 32-301 | 507 |
|  | merged & single reads | 11,162,444 | 50-586 | 3788 |

**Table S3.**

Comparison of the assembly of using different assembly software

|  | **SPAdes** | **Velvet** | **IDBA-UD** |
| --- | --- | --- | --- |
| No.contigs | 90 | 152 | 140 |
| Largest.contigs(bp) | 1,392,957 | 1,007,762 | 1,354,876 |
| Average.length(bp) | 146,146 | 86,675 | 93,886 |
| Total.size(Mb) | 13.15 | 13.17 | 13.14 |
| N10 | 1,392,957 | 769,344 | 1,354,876 |
| N50 | 653,939 | 270,470 | 522,380 |
| N90 | 117,109 | 47,909 | 56,675 |
| Note: the statistics is calculated after filtering out the fragments with length shorter than 500 bp , read coverage lower than 5. | | | |

**Table S4.**

Statistics of annotation results for two heterotrophic bacteria in coculture

|  | ***Pseudomonas stutzeri* TAIHU** | ***Mesorhizobium* sp*.* TAIHU** |
| --- | --- | --- |
| No.Contigs | 38 | 18 |
| Size (bp) | 4,830,778 | 4,926,263 |
| GC (%) | 63.63 | 63.43 |
| CDS | 4401 | 4806 |
| No. RNA | 55 | 49 |

**Table S5.**

The average nucleotide identity (ANI) value and estimated DNA-DNA hybridization (DDH) values between the two bacteria in coculture and other known strains in *Pseudomonas stutzeri*.

| **Reference genome** | **DDH** | **Distance** | **Prob. DDH >= 70%** | **ANI** |
| --- | --- | --- | --- | --- |
| *Pseudomonas stutzeri* NT0124_3069 | 87.3 | 0.0151 | 94.78 | 0.9857 |
| *Pseudomonas stutzeri* SLG510A3-8_3072 | 86.4 | 0.0161 | 94.4 | 0.9854 |
| *Pseudomonas stutzeri* T13_435 | 85.9 | 0.0167 | 94.14 | 0.9845 |
| *Pseudomonas stutzeri* DSM_4166_176 | 83.5 | 0.0193 | 92.92 | 0.9829 |
| *Pseudomonas stutzeri* XLDN-R_384 | 83.3 | 0.0195 | 92.8 | 0.9818 |
| *Pseudomonas stutzeri* ATCC_17588 | 80.9 | 0.0224 | 91.22 | 0.9805 |
| *Pseudomonas stutzeri* A1501_123 | 79.7 | 0.0237 | 90.38 | 0.979 |
| *Pseudomonas stutzeri* B1SMN1_333 | 79.4 | 0.0241 | 90.09 | 0.9788 |
| *Pseudomonas stutzeri* BAL361_3068 | 32 | 0.132 | 0.23 | 0.8833 |
| *Pseudomonas stutzeri* NF13_331 | 32 | 0.1317 | 0.23 | 0.8831 |
| *Pseudomonas stutzeri* ST-9_3071 | 31.7 | 0.133 | 0.21 | 0.882 |
| *Pseudomonas stutzeri* 19SMN4_3066 | 31.5 | 0.1343 | 0.19 | 0.8814 |
| *Pseudomonas stutzeri* RCH2_193 | 31.5 | 0.1341 | 0.2 | 0.8805 |
| *Pseudomonas stutzeri* CCUG_29243_177 | 31.2 | 0.136 | 0.17 | 0.8791 |
| *Pseudomonas stutzeri* KOS6_391 | 30.1 | 0.1415 | 0.11 | 0.8765 |
| *Pseudomonas stutzeri* CCUG_16156_332 | 29.6 | 0.1441 | 0.09 | 0.8747 |
| *Pseudomonas stutzeri* TS44_432 | 26.5 | 0.1633 | 0.02 | 0.8629 |
| *Pseudomonas stutzeri* DSM_10701_178 | 24.1 | 0.1812 | 0.01 | 0.8577 |
| *Pseudomonas stutzeri* SDM-LAC_387 | 22.4 | 0.1959 | 0 | 0.8499 |
| *Pseudomonas stutzeri* NT0128_3070 | 22.3 | 0.1962 | 0 | 0.8486 |
| *Pseudomonas stutzeri* 28a24_3065 | 22.1 | 0.1981 | 0 | 0.8477 |

**Table S6.**

The average nucleotide identity (ANI) value and estimated DNA-DNA hybridization (DDH) values between the two bacteria in coculture and other known strains in *Mesorhizobium* genus.

| **Reference genome** | **DDH** | **Distance** | **Prob. DDH >= 70%** | **ANI** |
| --- | --- | --- | --- | --- |
| *Mesorhizobium* sp. ORS3359 | 20.8 | 0.2107 | 0 | 0.8378 |
| *Mesorhizobium* sp. URHC0008 | 20.8 | 0.211 | 0 | 0.8361 |
| *Mesorhizobium* sp. URHA0056 | 20.7 | 0.2124 | 0 | 0.8366 |
| *Mesorhizobium* sp. L48C026A00 | 20.6 | 0.2132 | 0 | 0.8369 |
| *Mesorhizobium* sp. LNHC221B00 | 20.6 | 0.2133 | 0 | 0.8357 |
| *Mesorhizobium* sp. LNHC232B00 | 20.6 | 0.2134 | 0 | 0.8357 |
| *Mesorhizobium* sp. LNHC252B00 | 20.6 | 0.2135 | 0 | 0.8364 |
| *Mesorhizobium* sp. ORS3324 | 20.6 | 0.213 | 0 | 0.8375 |
| *Mesorhizobium* metallidurans STM | 20.5 | 0.2144 | 0 | 0.8367 |
| *Mesorhizobium* sp. 1M-11 | 20.5 | 0.2148 | 0 | 0.8346 |
| *Mesorhizobium* sp. LSJC280B00 | 20.5 | 0.2148 | 0 | 0.8351 |
| *Mesorhizobium* ciceri CMG6 | 20.4 | 0.2157 | 0 | 0.8368 |
| *Mesorhizobium* loti CJ3sym | 20.4 | 0.2154 | 0 | 0.8359 |
| *Mesorhizobium* loti MAFF303099 | 20.4 | 0.2152 | 0 | 0.8366 |
| *Mesorhizobium* loti R7A | 20.4 | 0.2157 | 0 | 0.8371 |
| *Mesorhizobium* loti USDA | 20.4 | 0.2158 | 0 | 0.8381 |
| *Mesorhizobium* sp. F7 | 20.4 | 0.2159 | 0 | 0.8375 |
| *Mesorhizobium* sp. L2C084A000 | 20.4 | 0.2151 | 0 | 0.836 |
| *Mesorhizobium* sp. NBIMC_P2-C1 | 20.4 | 0.2158 | 0 | 0.8364 |
| *Mesorhizobium* sp. NBIMC_P2-C2 | 20.4 | 0.2156 | 0 | 0.8366 |
| *Mesorhizobium* sp. NBIMC_P2-C3 | 20.4 | 0.2157 | 0 | 0.8363 |
| *Mesorhizobium* sp. WSM3626 | 20.4 | 0.2154 | 0 | 0.836 |
| *Mesorhizobium* amorphae CCNWGS0123 | 20.3 | 0.2169 | 0 | 0.8366 |
| *Mesorhizobium* ciceri biovar | 20.3 | 0.2164 | 0 | 0.8365 |
| *Mesorhizobium* ciceri WSM4083 | 20.3 | 0.2166 | 0 | 0.8357 |
| *Mesorhizobium* loti NZP2037 | 20.3 | 0.2168 | 0 | 0.8373 |
| *Mesorhizobium* plurifarium genome | 20.3 | 0.2161 | 0 | 0.838 |
| *Mesorhizobium* sp. LNHC229A00 | 20.3 | 0.2161 | 0 | 0.8346 |
| *Mesorhizobium* sp. LNJC384A00 | 20.3 | 0.2169 | 0 | 0.8354 |
| *Mesorhizobium* sp. LNJC386A00 | 20.3 | 0.2169 | 0 | 0.8352 |
| *Mesorhizobium* sp. LNJC398B00 | 20.3 | 0.2167 | 0 | 0.8352 |
| *Mesorhizobium* sp. LSHC424B00 | 20.3 | 0.217 | 0 | 0.8354 |
| *Mesorhizobium* sp. NBIMC P2-C4 | 20.3 | 0.2161 | 0 | 0.8363 |
| *Mesorhizobium* sp. LSJC265A00 | 20.3 | 0.2168 | 0 | 0.8347 |
| **Reference genome** | **DDH** | **Distance** | **Prob. DDH >= 70%** | **ANI** |
| *Mesorhizobium* sp. SOD10 | 20.3 | 0.2164 | 0 | 0.8376 |
| *Mesorhizobium* sp. URHB0007 | 20.3 | 0.2168 | 0 | 0.8351 |
| *Mesorhizobium* sp. WSM1293 | 20.3 | 0.2168 | 0 | 0.8369 |
| *Mesorhizobium* sp. WSM2561 | 20.3 | 0.216 | 0 | 0.8363 |
| *Mesorhizobium* sp. WSM3224 | 20.3 | 0.2163 | 0 | 0.8367 |
| *Mesorhizobium* ciceri ca181 | 20.2 | 0.2175 | 0 | 0.8345 |
| *Mesorhizobium* loti R88b | 20.2 | 0.2173 | 0 | 0.8356 |
| *Mesorhizobium* sp. L2C067A000 | 20.2 | 0.2179 | 0 | 0.8358 |
| *Mesorhizobium* sp. L2C089B000 | 20.2 | 0.2181 | 0 | 0.8358 |
| *Mesorhizobium* sp. L103C105A0 | 20.2 | 0.2178 | 0 | 0.8361 |
| *Mesorhizobium* sp. L103C119B0 | 20.2 | 0.2175 | 0 | 0.8351 |
| *Mesorhizobium* sp. L103C120A0 | 20.2 | 0.2181 | 0 | 0.8354 |
| *Mesorhizobium* sp. L103C565B0 | 20.2 | 0.2172 | 0 | 0.8348 |
| *Mesorhizobium* sp. LNJC372A00 | 20.2 | 0.218 | 0 | 0.8354 |
| *Mesorhizobium* sp. LNJC374B00 | 20.2 | 0.218 | 0 | 0.8352 |
| *Mesorhizobium* sp. LNJC380A00 | 20.2 | 0.2178 | 0 | 0.8355 |
| *Mesorhizobium* sp. LNJC395A00 | 20.2 | 0.218 | 0 | 0.8357 |
| *Mesorhizobium* sp. LNJC399B00 | 20.2 | 0.2176 | 0 | 0.8345 |
| *Mesorhizobium* sp. LNJC403B00 | 20.2 | 0.218 | 0 | 0.8357 |
| *Mesorhizobium* sp. LSHC414A00 | 20.2 | 0.2181 | 0 | 0.8354 |
| *Mesorhizobium* sp. LSHC416B00 | 20.2 | 0.2179 | 0 | 0.8354 |
| *Mesorhizobium* sp. LSHC420B00 | 20.2 | 0.2179 | 0 | 0.8357 |
| *Mesorhizobium* sp. LSJC255A00 | 20.2 | 0.2179 | 0 | 0.8348 |
| *Mesorhizobium* sp. LSJC269B00 | 20.2 | 0.2174 | 0 | 0.8355 |
| *Mesorhizobium* sp. LSJC277A00 | 20.2 | 0.2173 | 0 | 0.8352 |
| *Mesorhizobium* sp. LSJC285A00 | 20.2 | 0.2173 | 0 | 0.835 |
| *Mesorhizobium* alhagi CCNWXJ12-2 | 20.1 | 0.2181 | 0 | 0.8363 |
| *Mesorhizobium* sp. L2C054A000 | 20.1 | 0.2189 | 0 | 0.8358 |
| *Mesorhizobium* sp. LNHC209A00 | 20.1 | 0.2184 | 0 | 0.8358 |
| *Mesorhizobium* sp. LNHC220B00 | 20.1 | 0.2188 | 0 | 0.8348 |
| *Mesorhizobium* sp. LNJC391B00 | 20.1 | 0.219 | 0 | 0.8358 |
| *Mesorhizobium* sp. LNJC394B00 | 20.1 | 0.2188 | 0 | 0.8351 |
| *Mesorhizobium* sp. LNJC405B00 | 20.1 | 0.2185 | 0 | 0.8363 |
| *Mesorhizobium* sp. LSHC422A00 | 20.1 | 0.2191 | 0 | 0.8358 |
| *Mesorhizobium* sp. LSHC426A00 | 20.1 | 0.2181 | 0 | 0.8352 |
| *Mesorhizobium* sp. LSHC432A00 | 20.1 | 0.2186 | 0 | 0.8353 |
| *Mesorhizobium* sp. LSHC440A00 | 20.1 | 0.2182 | 0 | 0.8347 |
| *Mesorhizobium* sp. LSHC440B00 | 20.1 | 0.2185 | 0 | 0.8352 |
| *Mesorhizobium* sp. L103C131B0 | 20 | 0.2195 | 0 | 0.8353 |
| *Mesorhizobium* sp. LSJC264A00 | 20 | 0.2201 | 0 | 0.8351 |
| *Mesorhizobium* sp. LSHC412B00 | 20 | 0.2203 | 0 | 0.8351 |
| **Reference genome** | **DDH** | **Distance** | **Prob. DDH >= 70%** | **ANI** |
| *Mesorhizobium* sp. LSJC268A00 | 20 | 0.2197 | 0 | 0.8359 |
| *Mesorhizobium* sp. L2C066B000 | 19.9 | 0.2204 | 0 | 0.8347 |
| *Mesorhizobium* sp. L2C085B000 | 19.9 | 0.2204 | 0 | 0.8355 |
| *Mesorhizobium* sp. UASWS1009 | 19 | 0.2312 | 0 | 0.8298 |

**Table S7.**

The scaffold lists of two heterotrophic bacteria in coculture sample.

| **Bacteria name** | **Scaffold list** |
| --- | --- |
| *Pseudomonas stutzeri* TAIHU | NODE_56_length_8583_cov_83.8117_ID_15315 |
| *Pseudomonas stutzeri* TAIHU | NODE_57_length_5602_cov_35.6493_ID_15317 |
| *Pseudomonas stutzeri* TAIHU | NODE_58_length_5518_cov_214.395_ID_15319 |
| *Pseudomonas stutzeri* TAIHU | NODE_65_length_2607_cov_188.879_ID_15333 |
| *Pseudomonas stutzeri* TAIHU | NODE_67_length_1919_cov_106.873_ID_15337 |
| *Pseudomonas stutzeri* TAIHU | NODE_72_length_1608_cov_48.4315_ID_15347 |
| *Pseudomonas stutzeri* TAIHU | NODE_73_length_1513_cov_67.1494_ID_15349 |
| *Pseudomonas stutzeri* TAIHU | NODE_80_length_1098_cov_757.082_ID_15363 |
| *Pseudomonas stutzeri* TAIHU | NODE_81_length_1066_cov_271.833_ID_15365 |
| *Pseudomonas stutzeri* TAIHU | NODE_82_length_1050_cov_144.541_ID_15367 |
| *Pseudomonas stutzeri* TAIHU | NODE_85_length_950_cov_153.287_ID_15373 |
| *Pseudomonas stutzeri* TAIHU | NODE_89_length_663_cov_77.2015_ID_15381 |
| *Pseudomonas stutzeri* TAIHU | NODE_90_length_663_cov_98.1679_ID_15383 |
| *Mesorhizobium* sp. TAIHU | NODE_1_length_1392957_cov_72.3496_ID_15205 |
| *Mesorhizobium* sp. TAIHU | NODE_2_length_1200254_cov_75.3237_ID_15207 |
| *Mesorhizobium* sp. TAIHU | NODE_3_length_1079102_cov_76.0324_ID_15209 |
| *Mesorhizobium* sp. TAIHU | NODE_8_length_630050_cov_79.5234_ID_15219 |
| *Mesorhizobium* sp. TAIHU | NODE_16_length_274477_cov_63.8418_ID_15235 |
| *Mesorhizobium* sp. TAIHU | NODE_18_length_218224_cov_81.4756_ID_15239 |
| *Mesorhizobium* sp. TAIHU | NODE_35_length_37734_cov_83.7793_ID_15273 |
| *Mesorhizobium* sp. TAIHU | NODE_41_length_31210_cov_63.1949_ID_15285 |
| *Mesorhizobium* sp. TAIHU | NODE_45_length_16333_cov_62.8238_ID_15293 |
| *Mesorhizobium* sp. TAIHU | NODE_47_length_15157_cov_74.3488_ID_15297 |
| *Mesorhizobium* sp. TAIHU | NODE_50_length_14559_cov_62.494_ID_15303 |
| *Mesorhizobium* sp. TAIHU | NODE_54_length_8887_cov_69.6313_ID_15311 |
| *Mesorhizobium* sp. TAIHU | NODE_66_length_2508_cov_228.109_ID_15335 |
| *Mesorhizobium* sp. TAIHU | NODE_71_length_1619_cov_70.6025_ID_15345 |
| *Mesorhizobium* sp. TAIHU | NODE_78_length_1131_cov_453.678_ID_15359 |
| *Mesorhizobium* sp. TAIHU | NODE_84_length_981_cov_157.053_ID_15371 |
| *Mesorhizobium* sp. TAIHU | NODE_94_length_540_cov_68.6126_ID_15391 |
| *Mesorhizobium* sp. TAIHU | NODE_95_length_540_cov_100.68_ID_15393 |
